# Five types of home-visit nursing agencies in Japan based on characteristics of service delivery: cluster analysis of three nationwide surveys

**DOI:** 10.1186/s12913-014-0644-8

**Published:** 2014-12-20

**Authors:** Sakiko Fukui, Noriko Yamamoto-Mitani, Junko Fujita

**Affiliations:** Department of Community Health Nursing, Graduate School of Nursing, The Japanese Red Cross University, 4-1-3 Hiroo, Shibuya-ku Tokyo, 150-0012 Japan; Department of Adult/Palliative Care Nursing, Graduate School of Medicine, The University of Tokyo, Tokyo, Japan

**Keywords:** Home-visit nursing agency, Categorization, Quality of care, Service delivery

## Abstract

**Background:**

The number of home-visit nursing agencies in Japan has greatly increased over the past 20 years since the Japanese government first introduced it in 1992 to meet the increased needs of home-bound elderly. Since then, home-visit nursing has come to serve for a variety of populations such as those with terminal-stage cancer, neurological diseases, psychiatric conditions, or children with chronic conditions; currently the number of agencies has reached 6,801 (as of April 2013). Yet little has been known about the details of their characteristics in terms of patient types or differences/similarities across regions. In this study, we developed a method to categorize home-visit nursing agencies throughout Japan based on their actual service delivery, in order to help improve healthcare policies allowing better services by those agencies.

**Methods:**

We performed a cluster analysis on data from two national databases (Survey of Institutions and Establishments for Long-term Care which is annually administered by the Ministry of Health, Labour and Welfare [dataset 1; n = 5,161] and Information Publication System for Long-term Care which is annually reported by home-visit nursing agencies to their respective prefectural governments [dataset 2; n = 4,400, matching rate to data set 1: 84.4%]), in addition to the results from our original nationwide Fax survey of the service delivery system of home-visit nursing agencies (dataset 3; n = 2,049 matching rate to data set 1: 39.3%).

**Results:**

The cluster analysis suggested five categories for home-visit nursing agencies based on the type of service delivery system. For deciding of these categories, we held 13 panel discussions with specialists to confirm that the categorization of the home-visit nursing agencies appropriately reflected their actual delivery systems. The five categories were: nurse-centered (560, 10.9%), rehabilitation-centered (211, 4.1%), psychiatric-centered (360, 7.0%), urban-centered (1,784, 34.5%), and rural-centered (2246, 43.5%).

**Conclusions:**

This five categorization system of home-visit nursing agencies could ensure appropriate healthcare policies that will allow agencies to provide better home-visit nursing services based on their patient and staff characteristics and regional needs. The findings would be valuable both in Japan as well as in other countries with rapidly growing aging populations.

## Background

The Ministry of Health, Labour and Welfare of Japan regarded 2012 as the start of “a community comprehensive care system” in each community throughout Japan, and started promoting and completing its system by 2025 for its super-aging society. Within this system, home-visit nursing is expected to play a major role in linking medical support and daily-life support.

Home-visit nursing agencies first opened in 1992, mainly for home-bound elderly. The Japanese government started promoting home-visit nursing services in medical insurance since 1994 and in long-term care insurance since 2000 [1]. Today, home-visit nursing services are covered mainly by long-term care insurance (approximately 75%); the remaining is covered by medical insurance [2]. Patients generally decide to use long-term care insurance prior to medical insurance for home-visit nursing services. On the other hand, patients who have diseases that are approved by the Ministry of Health, Labor and Welfare such as terminal-stage cancer and neurological diseases, or who are less than 39 years old, are regulated to use medical insurance [3].

During the last 20 years since the home-visit nursing system was started, there have been many positive revisions for home-visit nursing services with both medical and long-term care insurance by each insurance policy revision with the implementation of the fee-for-services policy [1-4]. A focus has been placed on securing a sufficient number of home-visit nursing agencies. As a result, various types of home-visit nursing agencies have been established to meet the needs of each region such as the provision of terminal-stage cancer, psychiatric, or neurological conditions. Currently the number of agencies has reached 6,801 (as of April 2013) [5].

Many patients despite their extensive medical care needs have been discharged and returned to their communities early. Thus agencies with nurses who can attend to their medical care needs are in great demand. In order to further promote the implementation of home-visit nursing services, a new system was introduced in 2014 to evaluate highly functional home-visit nursing agencies for patients with extensive medical care needs (e.g., patients in need of end-of-life care or intensive medical care). In this new system, home-visit nursing agencies were divided into three classifications (Classifications I, II, and General); highly functional agencies of Classification I and II were provided greater reimbursement from the government for services based on the number of such patients in the previous year [4]. As this system currently covers home-visit nursing services through medical insurance only (comprising approximately 25% of patients), a more general system of classification of all home-visit nursing services is needed in Japan to allow for more services to be covered by long-term care insurance.

In consideration of the healthcare situation, we need more appropriate healthcare policies for improved home-visit nursing services that adequately reflect the characteristics and the regional needs of each agency [6]. We thus need to better understand the characteristics of these agencies as well their patients they serve. In this study, we aimed to develop a new method to categorize home-visit nursing agencies throughout Japan based on the actual services delivered for application to revisions to healthcare policies in Japan.

## Methods

### Surveys

The actual service delivery systems and financial status of home-visit nursing agencies in Japan have been previously reported over a four-year period (2009-2012) [7-10]. In these previous studies, we randomly selected roughly half of all home-visit nursing agencies and analyzed their characteristics. This method accompanies selection bias, as the selected agencies were those that were willing to participate. Therefore, we also used data from databases which are updated annually by the national and prefectural governments. By incorporating these resources, we could more accurately determine the overall characteristics of home-visit nursing agencies in Japan.

In this study, we first acquired data from two national public databases the Survey of Institutions and Establishments for Long-term Care in 2011 and Information Publication System for Long-term Care in 2012, which was the most recent data from the Ministry of Health, Labour and Welfare during the investigation period (July to September 2013). To complement the data obtained from these two databases, we performed our own nationwide FAX survey of the service delivery system of the home-visit nursing agencies which could better reflect the actual situation of patients using these agencies in January 2014.

With these three datasets, we performed a cluster analysis to determine a categorization of home-visit nursing agencies based on characteristics related to service delivery. During the acquisition of data, 13 panel discussions between the research project members and specialists, i.e., home-visit nursing specialists, administrative officers, physicians, and researchers, were held to confirm whether the data could appropriately show the actual situations of home-visit nursing service delivery nationwide. As a result, we identified five distinct types of home-visit nursing agencies based on their actual service delivery.

### Survey items and participants

For the Survey of Institutions and Establishments for Long-term Care, the main body of the investigation was the Japanese Ministry of Health, Labour and Welfare. The survey was conducted in September 2011 on the 6,047 certified home-visit nursing agencies nationwide. Among the 5,410 agencies that responded to the survey (response rate: 89.5%), we selected 5,161 agencies (85.3%) to analyze in this study; agencies that lacked data on their service delivery system were excluded. The survey items included 1) the activities of the agency; 2) the main body of establishment of the agency, 3) any notification of additions for either long-term care insurance or medical insurance; 4) presence of satellite office(s); 5) service delivery system (during September of the year) including the days of operation; 6) patient characteristics (during September of the year) including number of patients who used long-term care insurance or medical insurance, their functional level, the number of home visits per month, and the number of patients who stopped receiving services due to death; 7) the number of staff members including nurses and rehabilitation staff; 8) the main job of the manager; and 9) any parallel establishment of care management agency [11].

For the Information Publication System for Long-term Care database, the main body for data collection was each prefectural government. In this system, agencies that have been established in each prefecture are required to report their latest information to the respective prefectural governments once a year. The prefectural governments collect this information and then provide it on a website, where patients can compare and appropriately select a home-care agency based on the offered services. Among the 6,293 home-visit nursing agencies nationwide, as of 2012, we selected 5,082 agencies for data analysis; 1,211 agencies were excluded due to the following reasons: 1) they were non-certified home-visit nursing agency (737 agencies), 2) they did not provide home-visit nursing services (226), 3) the agency opened on or after January 1, 2013 (86), 4) the converted number of full-time nursing care workers was less than 2.5 persons, which number is regularly required to set up an agency (100), 5) they had no medical facilities/physicians which received directions (49), and 6) they had no patients (13). The survey items included: 1) activities of the agency; 2) location and address of the agency; 3) information related to staff members, including nurses and rehabilitation staff; 4) detailed information about the services provided; 5) information about the corporate business; 6) approaches to ensure high quality services; 7) cooperation with other organizations; and 8) system for appropriate agency operation/management [11].

In addition, we performed our own FAX survey of 5,568 home-visit nursing agencies throughout Japan in January 2014. We received responses from 3,042 agencies (response rate: 54.6%). From those, we selected 2,904 agencies for the analysis; 109 agencies that lacked an office number and 29 agencies that opened in September 2012 or later were excluded. The research group members then selected nine survey items regarding the service delivery systems of the agencies which were not addressed in the other two national public databases; these items were: 1) services provided by the agency; 2) types of office numbers; 3) owned by a hospital or not; 4) days of operation including days of the week and holiday operations; 5) home-visit nursing provisions to ≥10 patients in a private nursing home and the ratio of patients in private nursing homes to all patients; 6) the number of collaborating hospitals/care management agencies; 7) the number of patients using long-term care/medical insurance and the number of patients diagnosed with terminal-stage cancer, psychiatric diseases, or intractable diseases; 8) the number of patient deaths during 2012 including the place of the death and the number of deaths from cancer; and 9) financial performance of the agency (profitable, balanced, unprofitable or unclear) [11].

In this study, we focused on “structure” within the three factors frequently used to evaluate quality of healthcare services: “structure”, “process”, and “outcome” [12]. We chose the survey items in this study based on items from six categories in our previous study that influenced the maintenance of standards of care and improvement of home-visit nursing services (operating structure, management of service, employment, patient utilization, financial condition, and regional cooperation) [1].

### Analysis methods

We combined the results of the three datasets: Survey of Institutions and Establishments for Long-term Care in 2011 (dataset 1), Information Publication System for Long-term Care in 2012 (dataset 2), and FAX survey in 2014 (dataset 3).

Next, we performed a cluster analysis using dataset 1 to identify similarities between agencies regarding their service delivery systems. Five types of home-visit nursing agencies were identified. These five types were then further analyzed by combining dataset 1 with datasets 2 and 3 to elucidate the detailed characteristics of each agency type. Finally, we confirmed the validity of the five types based on the results of the cluster analyses.

For all data analyses, we used SAS statistical software (version 9.2).

### Ethical consideration

This study was performed after obtaining approval from the ethical committee of the national association of home-visit nursing care (approval No: 20141). We requested the agencies to return the questionnaire after explaining the study objectives and details in the fax letter to request participation in the study, and that the return of the questionnaire would be considered as their agreement for participation in the study as for Fax survey.

## Results

### Overall characteristics of home-visit nursing agencies

The overall characteristics of the 5,161 home-visit nursing agencies included in our study are shown in Table 1. The majority of the agencies were established by hospital corporations (39.3%), followed by business corporations (26.9%), social welfare corporations (7.4%), incorporated associations/general incorporated foundations (5.6%), medical associations (5.1%), and nursing associations (2.7%). The average number of workers (converted based on a full-time work worker) in each agency was 6.0 ± 3.6 persons, which included nurses (4.7 ± 2.5) and rehabilitation staff (0.9 ± 2.2). Of the total staff, nurses comprised 69.3%, while rehabilitation staff and designated office workers comprised 22.7% and 7.4%, respectively.

**Table 1:** **Features of five types of home-visit nursing agencies in Japan categorized by cluster analysis from three nationwide surveys**

	**Total**	**Nurse centered**	**Rihabilitation centered**	**Psychiatric centered**	**Urban centered**	**Rural centered**
	**n = 5,161 (100.0%)**	**n = 560 (10.9%)**	**n = 211 (4.1%)**	**n = 360 (7.0%)**	**n = 1,784 (34.5%)**	**n = 2,246 (43.5%)**
**A) Operating structure**						
Main body of establishment						
Hospital corporation (a)	39%	30%	26%	43%	38%	43%
Medical association (a)	5%	10%	0%	2%	6%	4%
Nursing association (a)	3%	8%	0%	2%	3%	2%
Incorporated association/general incorporated foundation (a)	6%	11%	1%	6%	6%	4%
Social welfare corporation (a)	7%	7%	6%	7%	7%	8%
Business corporation (a)	27%	17%	64%	31%	27%	26%
Others (a)	13%	17%	2%	11%	14%	13%
Operating years (average)						
Operating years (b)	10.9	12.8	9.3	10.4	11.2	10.4
Parallel establishment of medical facility						
Clinics with no beds (c)	13%	13%	13%	5%	13%	13%
Clinics with beds (c)	4%	1%	2%	4%	4%	5%
Hospitals (c)	37%	39%	23%	58%	35%	37%
None/missing value (c)	47%	49%	64%	34%	49%	46%
Care management agency and branch agency						
Parallel establishment of care management agency (a)	54%	64%	41%	38%	57%	53%
Another post of a worker as care manager (a)	18%	32%	8%	11%	21%	15%
Establishment of branch agency (a)	4%	10%	9%	2%	3%	2%
**B) Management of service**						
Operation days						
Only weekdays (b)	44%	47%	55%	40%	44%	42%
Weekdays and Saturdays (b)	47%	43%	35%	53%	46%	48%
Weekdays, Saturdays, and Sundays (b)	10%	10%	10%	7%	10%	10%
Status of notification system						
Addition of emergency services delivery (a)	86%	98%	62%	68%	90%	84%
Addition of 24-hour service delivery (a)	77%	97%	53%	55%	83%	72%
Addition of intensive medical management (a)	92%	100%	77%	66%	97%	91%
Addition of end-of-life care (a)	85%	98%	61%	59%	90%	83%
Home-visit nursing care in private nursing homes						
Provisions of home-visit nursing services for patients in private nursing homes (c)	6%	7%	8%	11%	5%	6%
**C) Employment**						
Number of workers (average)						
Nurses - converted as full-time workers (a)	4.7	8.8	5.1	4.4	4.2	4
Rehabilitation staff - converted as full-time workers (a)	0.9	1.1	8.3	0.4	0.6	0.5
Ratio of rehabilitation staff to total workers (a)	23%	0%	83%	1%	3%	2%
**D) Patient utilization**						
Patients (average)						
Number of patients (a)	65.9	113.8	184	58.4	73	38.4
Facilities with patients using medical insurance who accounted for 50% or more (a)	7%	4%	2%	90%	1%	0%
Cancer at terminal stage (c)	2.6	5.8	2.3	1.3	2.6	1.9
Intractable disease (c)	6.7	11.1	21.1	5.1	6.5	4.4
Psychiatric disease (c)	5.2	4.8	2.2	50.7	2.7	1.7
Number of patients and home visits per worker (average)						
Patients per worker (a)	11.6	11.9	13.9	12.1	15.4	8.3
Patients of terminal-stage cancer care per worker (c)	0.5	0.6	0.2	0.2	0.5	0.4
Patients with intractable diseases per worker (c)	1.1	1.1	1.5	1	1.3	0.9
Patients with psychiatric diseases per worker (c)	1	0.4	0.2	9.7	0.6	0.4
Pediatric patients per worker (c)	0.2	0.2	0.3	0.4	0.2	0.1
Home visits per worker (a)	64.4	68.8	72.5	61.3	85.1	46.6
System to accept patients						
Home intravenous alimentation (b)	83%	98%	75%	56%	89%	78%
Mechanical ventilation (b)	72%	91%	77%	49%	77%	64%
Pain control with narcotic medication (b)	84%	97%	69%	55%	90%	80%
Number of patient deaths per year (average)						
Total number of patient deaths (c)	13.9	32.4	13.1	5.4	14.5	9.2
Number of patient deaths at home (c)	7.8	19.6	7	3.2	7.9	4.8
Number of patient deaths at a place other than home (c)	6.1	12.8	6.2	2.2	6.6	4.3
Rate of deaths at home (c)	62%	65%	59%	69%	62%	60%
Ratios related to death from cancer (average)						
Number of patients who died from cancer (c)	8.5	19	6.3	3.7	8.5	6.2
Ratio of cancer patients among patients who died (c)	45%	47%	30%	38%	44%	47%
Ratio of deaths at home of patients who died from cancer (c)	54%	59%	50%	52%	56%	51%
Ratio of deaths at home of patients who died due to a cause other than cancer (c)	46%	49%	37%	53%	47%	46%
**E) Financial condition**						
Financial performance of agency						
Profitable (c)	44%	62%	58%	35%	49%	34%
Balanced (c)	27%	19%	26%	22%	31%	27%
Unprofitable (c)	20%	14%	3%	25%	14%	28%
Unclear (c)	9%	6%	14%	18%	6%	11%
**F) Regional cooperation**						
Characteristics of location of the facility (cities, towns and villages)						
Population density median (b)	1122/km^2^	1234/km^2^	3538/km^2^	1157/km^2^	1992/km^2^	808/km^2^
Population aging rate average (b)	23.4	22.8	22.2	22.5	23.1	24
Directions and cooperation (average)						
Collaborative hospitals who received directions (b)	23	34	53	16	26	17
Collaborative physicians who received directions (b)	34.2	52.9	75.8	25.7	38.4	24
Collaborative care management agencies	18	25	39	12	20	13

(a): Data from the Survey of Institutions and Establishments for Long-term Care in 2011 (n = 5161).

(b): Data from the Information Publication System for Long-term Care in 2012 (n = 4,400, matching rate to data (a): 84.4%).

(c): Data from the a nationwide Fax survey in 2014 (n = 2,049 matching rate to data (a): 39.3%).

### Characteristics of home-visit nursing agencies by type

Using dataset 1 (n = 5,161), dataset 2 (n = 4,400; matching rate for 5,082 agencies from dataset 1: 84.4%) and dataset 3 (n = 2,049; matching rate for 2,904 agencies from dataset 1: 39.3%), we performed a cluster analysis to categorize home-visit nursing agencies based on their actual service delivery systems. We identified five different agency types and labeled them based on the main common characteristics; the latter two types are general non-featured types that were categorized based on location (population density). Table 1 shows detailed characteristics of each type.

I. Nurse centered (560 agencies, 10.9%): They have the highest average number of nurses of 8.8. 62.2% are financially stable (balanced). They have the highest ratio of notifications of additions (i.e., additional services, such as 24-hour and emergency services delivery) and of acceptance of various type of patients (i.e., home intravenous alimentation, mechanical ventilation, and pain control with narcotic medication); and highest average number of patients requiring terminal-stage cancer and of home deaths per year.
II. Rehabilitation centered (211, 4.1%): They have the highest average number of rehabilitation staff (8.3 persons). 58.1% are financially profitable; 64% are owned by business corporations. The agencies categorized of this type are located in the regions with the highest average population density. They have the highest rate of operating days that were weekdays only; highest average number of collaborative hospitals and physicians with directions, and collaborative care management agencies; and highest average number of patients with intractable diseases; and comparatively smaller ratio of notifications of additions.
III. Psychiatric centered (360, 7.0%): They have the highest average number of patients with psychiatric diseases (50.7 persons). Many are owned by hospitals. They have a comparatively low ratio of notifications of additions; highest ratio of home-visit nursing provision in private nursing homes; and highest ratio of agencies with ≥50% patients using medical insurance.
IV. Urban centered (1,784, 34.5%): This group is one general type based on location in regions with the second highest population density and no other major features on workers and patients. Approximately one third of all agencies is included in this type. They have the second highest ratio of notifications of additions, following the nurse-centered agencies; an average number of nurses (4.2) that is similar to the national average; and the highest average numbers of patients per worker (15.4) and home visits per worker (85.1).
V. Rural centered (2,246, 43.5%): This is one general type based on location in the lowest populated region and no other major features on workers and patients. 44% of all agencies is included in this type. They have an average number of nurses (4.0) which is similar to the national average; the highest rate of agencies that are financially unprofitable; and the lowest average numbers of patients per worker (8.3) and home visits per worker (46.6) compared to the other four types.

## Discussion

This study identified five distinct types of home-visit nursing agencies. The nurse-centered agencies accounted for approximately 10% of all agencies. This type of agency had a characteristically larger number of nurses than other agency types and less overall workers than rehabilitation-centered agencies. Since the number of nurses was high, the numbers of terminal-stage cancer patients (monthly average: 5.8 persons) and home deaths per year (19.6) were high. In general, these agencies accepted patients with a high level of medical dependence, frequently requiring services such as home intravenous alimentation, mechanical ventilation, and control of narcotic medication.

Nurse-centered agencies appear to be most similar to home-visit nursing services of Classification I in the latest revision of the medical insurance policy in 2014. Agencies of this type could expect to fulfill the standards for highly functional home-visit nursing agencies that can accept terminal care or high severity cases: ≥7 full-time nurses; ≥20 additions per year for home-visit nursing terminal care expense or terminal care; and ≥10 patients who are qualified and use medical insurance [4].

In contrast, the urban-centered agencies accounted for approximately one third of all agencies. The main characteristics of this type of agency are 4.2 full-time nurses, 14.5 patient deaths per year, and 7.9 patient deaths at home. Similar to the nurse-centered agencies, they have a relatively high acceptance of patients requiring medical dependence services (80-90%). In comparison, the 2014 revision of the medical insurance policy’s Classification II agencies are characterized as ≥5 full-time nurses, ≥15 additions per year for home-visit nursing terminal care expense or terminal care, and ≥7 patients who are qualified and use medical insurance. Thus, urban-centered agencies are expected to play a role as Classification II home-visit nursing agencies within the 2014 revision of the medical insurance policy [4].

On the other hand, the rehabilitation-centered agencies are distinct from other agency types. First of all, their services are mainly provided by rehabilitation staff in the home-visit nursing agency. These services were established as an operating model of home-visit nursing agency, and thus agencies of this type are increasing [7]. In this operating model, a business corporation establishes the agency in a region of extremely high population density, employs rehabilitation staff rather than nurses, and liaisons with more physicians and care managers. Notably, these agencies provided fewer 24-hour or emergency services and end-of-life care, limiting their services mainly to weekdays. With the rapid increase in elderly patients especially in urban areas [13], the need for such rehabilitation services will definitely increase in the future. A previous study has shown that a large number of total workers and nurses, which indicates the size of an agency, has been associated with financial stability of that agency [7]. However, our study showed that even if urban-centered agencies maintained high numbers of patients and home visits per worker (15.4 persons and 85.1 times, respectively), they still had lower financial stability than nurse- and rehabilitation-centered agencies. This suggests that the number and ratio of rehabilitation staff in an agency are important for maintaining the financial stability of the agency. Future discussion is thus necessary regarding the rehabilitation services component of home-visit nursing.

A new type of agency presented in this study is the psychiatric-centered agency. The characteristics of this type include ownership by a hospital, majority of psychiatric patients, a high ratio of patients with medical insurance, and a low ratio of emergency and 24-hour services. Although the reimbursement structure of home-visit nursing agencies for psychiatric patients was reviewed in the revision of medical treatment fees in 2012 [14], there is still insufficient information about the service delivery system by these psychiatric-centered agencies and more precise investigation will be needed.

Lastly, we identified the rural-centered type, making up the largest group of home-visit nursing agencies relative to population. To date, payment for home-visit nursing services has been uniform nationwide, with a base cost that is determined on a per-hour basis for long-term care insurance and a per-number-of-times basis for medical insurance. Comparing differences in regional characteristics, medical fees for home-visit nursing in certain regions, such as intermountain regions and isolated islands, are considered additional services (“additions”) [3]. However, rural-centered agencies although accounting for approximately 45% of all agencies in this study, were not localized in underpopulated areas, such as the aforementioned isolated islands and intermountain regions; their average population density is 808 persons/km^2^ (e.g., Tsukuba City in Ibaraki Prefecture, Choshi City in Chiba Prefecture, Hachinohe City in Aomori Prefecture, and Munakata City in Fukuoka Prefecture, population density: approximately 800 persons/km^2^). The home-visit nursing agencies in these regions that provided services with disregards to their financial performance tended to be unprofitable. In the future, an appropriate payment system to support the agencies of this type should be considered.

As a limitation of this study, no large difference was confirmed except for population density and numbers of patients and home visits per worker between the urban- and rural-centered agencies, although the agencies of these types accounted for approximately 80% of all agencies. Thus, the characteristics of these two types should be further examined. In addition, since the categorization of home-visit nursing agencies was performed with a focus placed on the “structure”, service delivery system, and not “process” and “outcome”, future studies could further sophisticate the categorization by focusing on these other two aspects of service. Factors of “process”, including a flexible predictive approach depending on the individual patients, a training system for such approach, and cooperation with other businesses, and factors related to “outcome”, such as changes in diseases and functional status of patients, satisfaction of patients, and cost, should also be incorporated to establish an appropriate system for further improvement of home-visit nursing services in Japan.

## Conclusion

The cluster analysis suggested five categories for home-visit nursing agencies based on the type of service delivery system: nurse-centered (560, 10.9%); rehabilitation-centered (211, 4.1%); psychiatric-centered (360, 7.0%); urban-centered (1,784, 34.5%); and rural-centered (2246, 43.5%).

This five categorization system of home-visit nursing agencies could ensure more appropriate healthcare policies for further improvement of home-visit nursing services by each agency in Japan could be applicable to other countries with rapidly growing aging populations.
